# A Case of Spontaneous Bilateral Direct Carotid-Cavernous Fistula

**DOI:** 10.7759/cureus.24634

**Published:** 2022-04-30

**Authors:** Francesco Pellegrini, Antonio Zappacosta, Daniele Cirone, Cristina Ciabattoni, Andrew G Lee

**Affiliations:** 1 Department of Ophthalmology, Santo Spirito Hospital, Pescara, ITA; 2 Department of Ophthalmology, Azienda Sanitaria Locale (ASL) Pescara, Pescara, ITA; 3 Department of Ophthalmology, Villa Anna Hospital, San Benedetto, ITA; 4 Department of Ophthalmology, Asur Marche Area Vasta 3, Macerata, ITA; 5 Department of Ophthalmology, Houston Methodist Hospital, Houston, USA

**Keywords:** superior ophthalmic veins, ophthalmoplegia, diplopia, proptosis, carotid-cavernous fistula

## Abstract

A 92-year-old female with poorly controlled systemic hypertension presented with bilateral eye redness, lid fullness, conjunctival chemosis, ophthalmoplegia, and ptosis for two days. A neuro-ophthalmic evaluation revealed bilateral proptosis, severe conjunctival chemosis and congestion, and an almost complete bilateral ophthalmoplegia with a complete right superior eyelid ptosis. Computed tomography (CT) scans demonstrated bilateral dilation of the superior ophthalmic veins, and a CT angiography (CTA) showed a direct high-flow carotid-cavernous fistula (CCF) with secondary extraocular muscle enlargement. Clinicians should be aware that a typical direct high-flow CCF, although usually occurs after trauma and unilaterally, can present spontaneously without trauma and bilaterally.

## Introduction

A carotid-cavernous sinus fistula is an abnormal communication between arteries and veins within the cavernous sinus. They are classified as direct or indirect depending upon the communication between the internal carotid artery and the cavernous sinus. Usually, direct fistulas are traumatic. We hereby present a rare case of bilateral direct nontraumatic carotid-cavernous fistula (CCF). The classification and clinical features of CCF are discussed in this study.

## Case presentation

**Figure 1:**
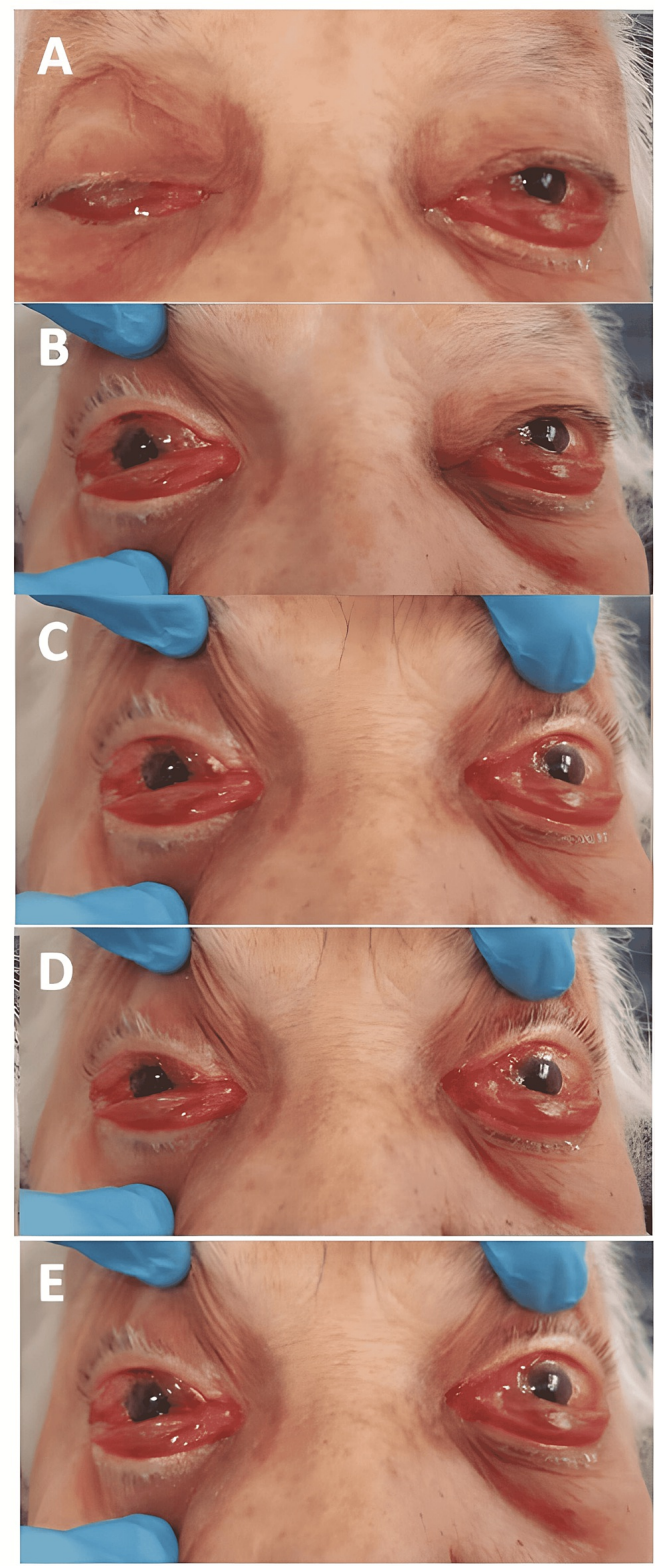
External appearance of the patient at presentation There is bilateral proptosis with right complete ptosis and global reduction of eye movements. (A) Primary position. (B) Upgaze. (C) Right gaze. (D) Left gaze. (E) Downgaze.

A 92-year-old female with poorly controlled systemic hypertension presented with bilateral eye redness, lid fullness, conjunctival chemosis, ophthalmoplegia, and ptosis for two days. She was treated with steroid ointment for “conjunctivitis.” The remainder of the past medical, surgical, social, family, and medication histories were non-contributory. A neuro-ophthalmic evaluation revealed bilateral proptosis, severe conjunctival chemosis and congestion, and an almost complete bilateral (OU) ophthalmoplegia with a complete right superior eyelid ptosis (Figure 1).

**Figure 2:**
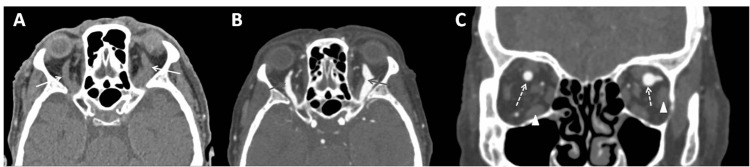
Computed tomography scan of the head (A) Axial computed tomography scan of head and orbit performed urgently shows bilaterally dilated superior ophthalmic veins (white arrows). (B) Axial computed tomography angiography confirms dilated superior ophthalmic veins (arrows) consistent with a diagnosis of direct carotid-cavernous fistula. (C) Coronal computed tomography angiography shows a mild enlargement of extraocular muscles (arrowhead) and dilated superior ophthalmic veins (dotted arrows).

The pupils were isochoric with no relative afferent pupillary defect (RAPD). The best-corrected visual acuity was 20/200 OU consistent with significant nuclear sclerosing cataracts OU. Intraocular pressure (IOP) was 21 mmHg OU with pulsatile mires on applanation tonometry. Fundus examination disclosed mild engorgement of retinal veins with no optic disc swelling. Computed tomography (CT) scans demonstrated bilateral dilation of the superior ophthalmic veins (SOV) (Figure 2). CT angiography (CTA) showed a direct carotid-cavernous high-flow fistula with secondary extraocular muscle enlargement (Figure 2). The patient chose to be treated in a nearby hospital and was lost on follow-up.

## Discussion

A CCF is an abnormal communication between arteries and veins within the cavernous sinus [1]. Direct CCFs (Barrow type A) are usually high-flow fistulas and represent direct communication between the internal carotid artery (ICA) and the cavernous sinus. Direct CCFs are usually post-traumatic but may be caused by the rupture of an intracavernous aneurysm or Ehlers-Danlos syndrome type IV or may be iatrogenic. Indirect (or dural) CCF (Barrow types B, C, and D) are usually low-flow fistulas that consist of communication between the cavernous sinus and cavernous arterial branches of the internal (B) or external (C) carotid arteries or both (D) [2].

Ophthalmic complications of CCF include secondary glaucoma, retinal vein and artery occlusion, anterior segment ischemia, serous retinal detachment, diplopia, and exposure keratopathy [3]. Thrombosis of the cavernous sinus, thyroid-associated ophthalmopathy, orbital neoplasms, and inflammatory pseudotumor of the orbit should also be considered in the differential diagnosis.

Bilateral spontaneous CCF, however, are rare and mostly indirect [4]. Al-Mufti et al. [5] reported a total of 26 bilateral CCF since 1963. Khan et al. updated the review and reported only 35 total cases of spontaneous, nontraumatic bilateral CCF [6]. Previously, the treatment for direct CCFs was limited to observation or trapping of the fistula by ligating the cervical ICA proximal to the fistula or occlusion of the common carotid artery or ICA. Those treatments could result in a cerebral ischemic or embolic event [7]. Now, the ICA almost always can be preserved, and a transarterial approach via the ICA is the most commonly used [8].

On the contrary, up to 70% of indirect CCFs close spontaneously. Other treatment options include compression of the ipsilateral ICA or SOV, stereotactic radiosurgery, and endovascular intervention [9]. Spontaneous closure of indirect CCFs may initially be associated with exacerbation of symptoms and signs.

## Conclusions

Clinicians should be aware that a typical direct high-flow CCFs, although usually occurs after trauma and unilaterally, can present spontaneously without trauma and bilaterally. Spontaneous direct CCFs should thus be considered in patients presenting with acute changes in vision, headache, and proptosis regardless of the history of trauma. The distinctive radiographic sign on CT or MRI is dilation of the SOV. Standard catheter angiography is typically necessary both for diagnosis and treatment with endovascular embolization.
